# Community stakeholder perspectives on the socio‐spatial rights of people living with dementia and caregivers

**DOI:** 10.1002/alz70858_098282

**Published:** 2025-12-25

**Authors:** Carmela Leone, Rachel Winterton, Irene Darmadi Blackberry

**Affiliations:** ^1^ John Richards Centre for Rural Ageing Research, La Trobe University, Bendigo, VIC, Australia; ^2^ John Richards Centre for Rural Ageing Research, Bendigo, VIC, Australia; ^3^ Care Economy Research Institute, La Trobe University, Albury‐Wodonga, VIC, Australia

## Abstract

**Background:**

The majority of people living with dementia and their caregivers live in their communities where, due to largely inaccessible public spaces and activities, they are at risk of experiencing socio‐spatial exclusion. A lack of access to the built environment impacts their opportunities for social connection, their potential for social health, and the achievement of health equity. To address the socio‐spatial exclusion of people living with dementia and caregivers, dementia‐friendly communities have been established worldwide. Dementia‐friendly communities often consider the rights of people living with dementia and caregivers; however, few are considered genuine rights‐based environments, and none specifically address socio‐spatial rights. This has implications for planning policy and practice in terms of ensuring socio‐spatial justice for people living with dementia and their caregivers.

**Method:**

Using a rights‐based conceptual framework, this study aimed to identify community stakeholder perspectives on the rights of people living with dementia and carergivers to participate in decision‐making, to be included in, and to access public spaces and social activities. Semi‐structured interviews were conducted with fifteen representatives of community stakeholder organisations and groups from a large non‐metropolitan region in Victoria, Australia.

**Result:**

Findings identified that socio‐spatial rights are facilitated by healthcare organisations and community groups which acknowledge the personhood of people living with dementia. The facilitation of socio‐spatial rights, however, is impeded by the absence of people living with dementia from local government decision‐making processes. Stigma associated with dementia was also identified as a barrier to achieving socio‐spatial justice, as were local government priorities. Not recognising dementia as a disability, a lack of dementia awareness, and the absence of dementia‐inclusive legislation and policy were also impediments.

**Conclusion:**

The recognition of dementia as a disability, dementia education, and dementia‐inclusive legislation and policies are critical to establishing genuine rights‐based dementia‐friendly communities, where socio‐spatial justice, social health and health equity can be achieved.

